# Effect of Cost-Related Medication Non-adherence Among Older Adults With Medication Therapy Management

**DOI:** 10.3389/fmed.2021.670034

**Published:** 2021-06-17

**Authors:** Weiwei Zhang, Gang Lv, Xiaomo Xiong, Minghui Li

**Affiliations:** ^1^Department of Clinical Pharmacy, Beijing Tsinghua Changgung Hospital, School of Clinical Medicine, Tsinghua University, Beijing, China; ^2^Department of General Surgery, The First Medical Center of Chinese PLA General Hospital, Beijing, China; ^3^Department of Clinical Pharmacy and Outcomes Sciences, College of Pharmacy, University of South Carolina, Columbia, SC, United States; ^4^Department of Clinical Pharmacy and Translational Science, University of Tennessee Health Science Center, Memphis, TN, United States

**Keywords:** medication therapy management, pharmacist, cost-related medication non-adherence, Medicare beneficiaries, Medicare part D

## Abstract

**Background:** Medication therapy management (MTM) was established by the Center for Medicare and Medicaid Services (CMS) with the aim to improve medication adherence. However, the national prevalence of cost-related medication non-adherence (CRN) is still unknown and there is a literature gap in the association between MTM services and CRN.

**Methods:** A cross-sectional study was conducted. A nationally representative study sample from Medicare Current Beneficiary Surveys (MCBS) was used. Survey sampling weights were applied for national estimates of CRN. Weighted multivariable logistic regressions controlling for covariates were conducted to investigate the effect of the MTM on the CRN.

**Results:** The study identified 1,549 MTM-eligible beneficiaries. The prevalence of CRN was higher in MTM-eligible individuals than in non-MTM eligible individuals (24.14 vs. 13.44%; *P* < 0.001). According to the results of multivariable logistic regressions, we found that MTM eligibility was significantly associated with a higher prevalence of CRN (OR: 1.59; 95% CI: 1.28–1.96). Additionally, some other variables such as health status, with or without low-income subsidy are also associated with CRN.

**Conclusions:** Our findings suggest that the prevalence of CRN in MTM-eligible beneficiaries was higher than in non-MTM eligible beneficiaries. Further studies with the longitudinal design are warranted to clarify the relationship between MTM and CRN. Alternative strategies to improve CRN should be considered in future Medicare Part D Enhanced MTM Models.

## Introduction

Since 2006, the Centers for Medicare and Medicaid Services (CMS) in the U.S. have required health plans for Medicare prescription drug benefit (Part D) to provide medication therapy management (MTM) service for Medicare eligible beneficiaries under the Medicare Modernization Act of 2003 (1). MTM services include providing education, improving adherence, and detecting adverse drug events and medication misuse (2). By providing these services, pharmacists can help eligible enrollees avoid drug-related problems and achieve desired clinical benefits of medications (3). There are five core elements in the MTM service module, including medication therapy review (MTR), personal medication record (PMR), medication-related action plan (MAP), intervention and/or referral, and documentation and follow-up (4, 5). Several studies have demonstrated that pharmacist-provided MTM services improve health outcomes and medication adherence (6–8). A recent systematic review reported that MTM programs might be able to reduce medication non-adherence and lower health care costs, but the evidence was insufficient due to inconsistency and imprecision that stem in part from underlying heterogeneity in populations and interventions (9). In addition, several studies have shown that some MTM programs were not able to improve medication non-adherence (10, 11). The association between MTM and medication non-adherence has not been fully confirmed.

An important type of medication non-adherence is cost-related medication non-adherence (CRN), which is specifically defined as behavior when patients take medication less than as prescribed due to costs (12). CRN is associated with serious health consequences, including decrements in self-reported health status, increased hospital admissions and death (13, 14). It has become a focus on policy research in understanding if an increase in insurance coverage through health policy can lead to the reduction of access barriers (15, 16). Early evaluations of Part D indicated modest nationwide reduction in CRN in 2006 and 2007 (15, 17). As an essential strategy to optimize medication use for Medicare Part D beneficiaries, MTM was assumed to reduce CRN among these beneficiaries. However, little is known about the prevalence of CRN among Medicare beneficiaries of the U.S. In addition, there is a literature gap in the association between MTM services and CRN among Medicare beneficiaries. We, therefore, conduct a study to provide national estimates of the prevalence of CRN among Medicare beneficiaries and identify the association between MTM services and CRN. We hypothesized that MTM-eligible beneficiaries will show lower CRN compared with non MTM-eligible beneficiaries.

## Materials and Methods

### Data and Study Design

This research was a cross-sectional study. Data from Medicare Current Beneficiary Surveys (MCBS) 2012 was used for this study. MCBS is a nationally representative sample of the Medicare population administrated by the Centers for Medicare and Medicaid Services (CMS). MCBS aim to determine spending and source of payment for services used by Medicare beneficiaries, relate coverage to sources of payment, and trace outcomes related to eligibility and impacts of the Medicare program. The MCBS sample is selected from Medicare Administrative Registration (MAE) data. A panel design is used for selection and each beneficiary selected is interviewed 12 times up to 4 years. The survey of the MCBS collects information on beneficiaries' demographic characteristics, insurance, health status, and the healthcare utilization and costs, and the information is merged with Medicare claims. The data in the MCBS are cleaned and structured. For participants who are not able to conduct in-person interviews because of unconsciousness, respondents can answer the survey questions on their behalf.

### Measurement

Medicare beneficiaries eligible for MTM in this study are Part D enrollees with at least two chronic diseases, taking at least three Part D drugs, and those who are likely to exceed a predetermined cost threshold of $3,100 according the MTM eligibility criteria (18, 19).

The outcome for this study was the CRN. Because there is no survey question specific to the CRN in the MCBS, we constructed a summary indicator of CRN for analysis based on references (15, 17, 20). Specifically, participants were identified as having the CRN if they answered “yes” to any of the following MCBS prompts: “decide not to fill or refill a prescription because it was too expensive,” “skipped doses to make the medicine last longer,” “taken smaller doses of a medicine to make the medicine last longer” and “spent less money on food, heat or other basic needs so that you would have money for medicine.” These questions were not specific to a particular class of medications.

To investigate the independent effect of MTM eligibility on CRN in the multivariate logistic regression models, we summarized potential confounders based on the literature (21–24). Potential confounders included demographic characteristics: age (18–64, 65–74, 75–84, 85+), gender (male, female), race (non-Hispanic white, non-Hispanic black, Hispanic, other), education (less than high school, high school graduate, some college, college graduate), marital status (married, widowed, single), residence (non-metropolitan, metropolitan), census region (Northeast, Midwest, South West); socio-economic factors: annual income (<$10,000, $10,001–20,000, $20,001–40,000, ≥ $40,000), low-income subsidy (LIS) (yes, no); and health behavior and physical health factors: smoking (no, former, current), health status (excellent, very good or good, fair or poor), body mass index (BMI) (underweight, normal, overweight, obese), and activities of daily living (ADL) (0, 1–2, 3+). They can be measured in the MCBS.

### Analysis

Survey sampling weights were applied to obtain national estimates of CRN. The analyses correct variance estimates for the complex survey design of the MCBS. Descriptive statistics were used to characterize the overall sample subjects. Chi-square or Fisher's exact test, whenever appropriate, was used in the study for categorical variables. Initial analyses focused on differences between the MTM-eligible beneficiaries and the non-MTM-eligible beneficiaries in responders' demographic characteristics and CRN behaviors. Multivariate logistic regression models were fit within each group to determine the independent effect of MTM eligibility on CRN, controlling for participants' age, gender, race, income, education, comorbidities, health status, low-income subsidy, and insurance coverage. In the logistic regression, when defining a reference group, we sorted the variables from lowest to highest of the original data and defined the lowest group as the reference group. However, we used the age group “65–74” as the reference group because Medicare beneficiaries mainly consist of older adults aged 65 years or older. We used normal as the reference group other than underweight for BMI. Institutional Review Board approval was obtained for this study. all the statistical analyses were performed using SAS (version 9.4; SAS Institute, Inc., Cary, NC).

## Results

This study identified 1,549 MTM-eligible beneficiaries in the MCBS. Table 1 shows weighted percentages of respondents with CRN by individual characteristics. Overall, the prevalence of CRN among MTM-eligible beneficiaries was 24.14%, with the following predominant characteristics: female (61.54%), non-Hispanic whites (70.99%), metropolitan (75.15%), excellent, very good, or good health status (57.92%), and without LIS (52.13%). The prevalence of CRN was much lower among respondents of non-MTM-eligible beneficiaries than those MTM-eligible beneficiaries (13.44%), with the following predominant characteristics: non-Hispanic white (73.34%), metropolitan (77.26%), excellent, very good, or good health status (78.59%), low ADL score (66.65%) and without LIS (74.06%).

**Table 1:** Respondent characteristics of the study population.

**Characteristics**	**MTM-eligible**	**Non-MTM-eligible**	***P*-value**
	**Weighted (%)**	**Weighted (%)**	
CRN			<0.001
Yes	24.14	13.44	
No	75.86	86.56	
Age			<0.001
18–64	26.44	15.18	
65–74	34.50	43.46	
75–84	28.62	29.48	
85+	10.44	11.88	
Gender			0.015
Male	38.46	42.71	
Female	61.54	57.29	
Race			0.383
Non-hispanic white	70.99	73.34	
Non-hispanic black	10.27	10.24	
Hispanic	12.56	11.40	
Other	6.19	5.02	
Education			0.044
Less than high school	28.08	24.55	
High school graduate	26.85	28.23	
Some college	24.26	23.19	
College graduate	20.82	24.04	
Marital status			<0.001
Married	39.00	48.64	
Widowed	27.61	25.41	
Single	33.39	25.95	
Annual income			<0.001
<$10,000	20.33	12.66	
$10,001–20,000	33.93	28.42	
$20,001–40,000	25.76	31.74	
≥ $40,000	19.98	27.17	
Residence			0.241
Non-metropolitan	24.85	22.74	
Metropolitan	75.15	77.26	
Census region			0.028
Northeast	20.00	18.79	
Midwest	21.24	22.92	
South	39.66	35.48	
West	19.10	22.81	
Health status			<0.001
Excellent, very good, or good	57.92	78.59	
Fair or poor	42.08	21.41	
BMI			<0.001
Underweight	1.77	2.51	
Normal	25.46	33.38	
Overweight	33.16	36.68	
Obese	39.61	27.43	
Smoking			<0.001
No	36.65	42.56	
Former	45.76	43.38	
Current	17.58	14.06	
ADL			<0.001
0	47.57	66.65	
1–2	32.12	22.52	
3+	20.31	10.82	
LIS			<0.001
Yes	47.87	25.94	
No	52.13	74.06	

*MTM, medication therapy management; CRN, cost-related non-adherence; BMI, body mass index; ADL, activities of daily living; LIS, low-income subsidy*.

**Figure 1:**
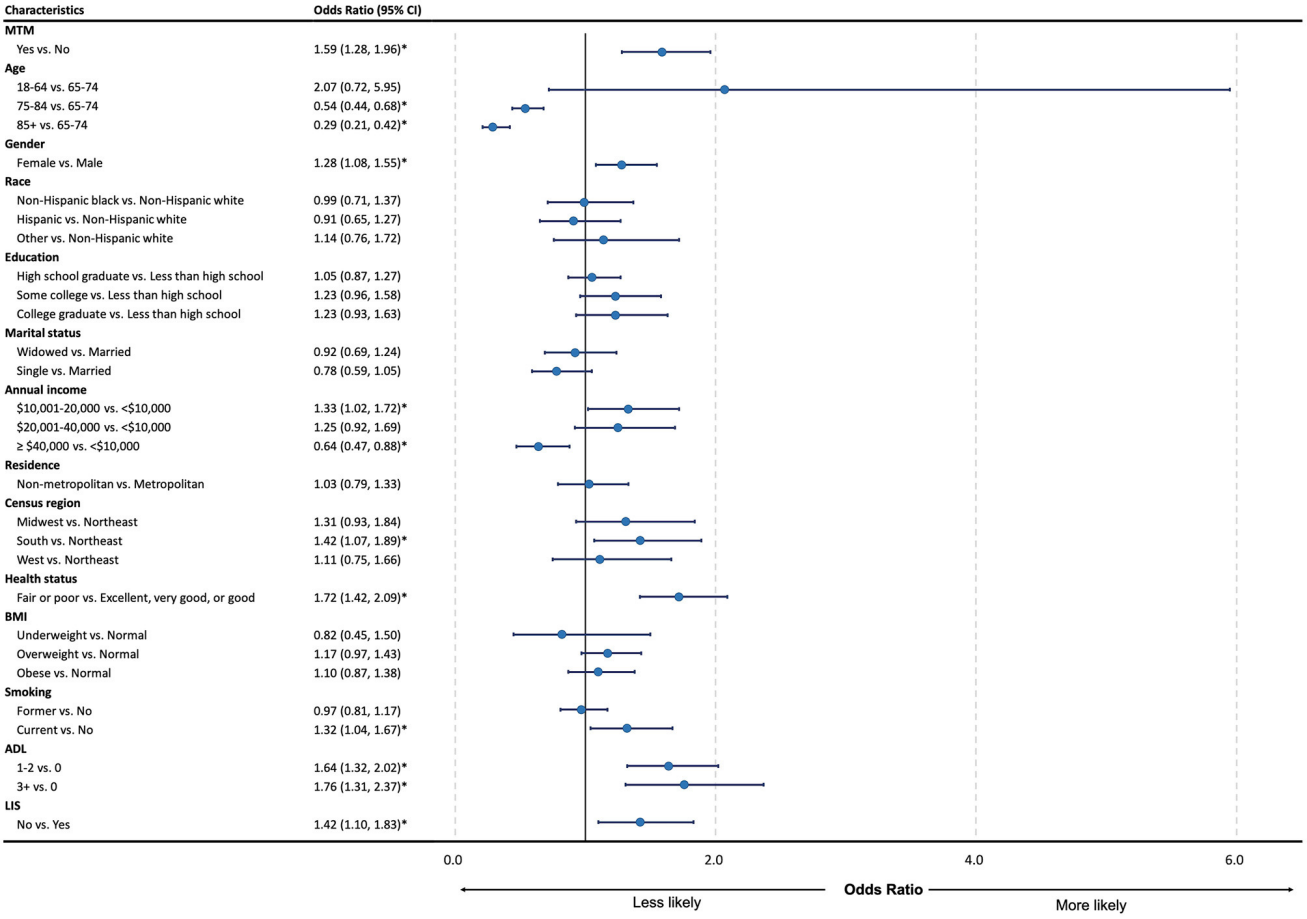
Forest plot for odds ratio and 95% CI of regression for cost-related medication non-adherence among the study group. MTM, medication therapy management; CRN, cost-related non-adherence; BMI, body mass index; ADL, activities of daily living; LIS, low-income subsidy; CI, confidence interval; LL, lower limit; UL, upper limit. ^*^P < 0.05.

Figure 1 shows that MTM eligibility was significantly associated with a higher prevalence of CRN [odds ratio (OR): 1.59; 95% confidence interval (CI): 1.28–1.96]. In addition to the MTM, we also found that the CRN was significantly associated with some demographic, socio-economic, health behavior, and physical health factors. Specifically, compared to Medicare beneficiaries aged between 65 and 74 years, those aged between 75 and 84 years (OR: 0.54; 95% CI: 0.44–0.68) and aged 85 years or older (OR: 0.29; 95% CI: 0.21–0.42) had a lower prevalence of the CRN. Females had a higher CRN prevalence than males (OR: 1.28; 95% CI: 1.08–0.55). Also, Medicare beneficiaries living in the South had a higher CRN prevalence compared to those living in the Northeast (OR: 1.42; 95% CI: 1.07–1.89). Compared with Medicare beneficiaries with an annual income of <10,000 those with an annual income between 10,001 and 20,000 had a higher CRN (OR: 1.33; 95% CI: 1.02–1.72), while those with an annual income of more than 40,000 had a lower CRN (OR: 0.64; 95% CI: 0.47–0.88). Meanwhile, people without the LIS had a higher prevalence of the CRN than those with (OR: 1.42; 95% CI:1.10–1.83). In terms of health behaviors, current smokers had a higher prevalence of the CRN compared to non-smoker (OR: 1.32; 95% CI:1.04–1.67). Medicare beneficiaries with excellent, very good, or good status had a lower prevalence of the CRN compared to those with fair or poor status (OR: 1.72; 95% CI:1.42–2.09), and Medicare beneficiaries with ADL had a higher prevalence of the CRN compared to those without ADL (1 ≤ ADL ≤ 2 vs. ADL = 0: OR: 1.64; 95% CI:1.32–2.02; ADL ≥ 3 vs. ADL = 0: OR: 1.76; 95% CI:1.31–2.37).

## Discussion

MTM aims to enhance patient understanding of appropriate drug use, increase patient adherence with prescribed drug therapies, reduce the risk of adverse events associated with drugs, and cut down on the need for other costly medical services (25). Previous studies demonstrated that MTM was optimal in saving cost, decreasing medication adverse events, and especially in improving adherence (6–8). In this study, we found that MTM eligibility was significantly associated with a higher prevalence of CRN.

The MTM-eligible beneficiaries were individuals with a higher estimated annual medical cost, many underlying disease state(s), and medications that are restricted by Medicare Part D prescription drug plans (26). These individuals with multiple medications tended to incur higher out-of-pocket medication costs and were reluctant to take medications and preferred to minimize medication intake (13, 27). That might be the main reason that contributed to higher CRN in the MTM-eligible beneficiaries. Though some previous studies showed the effect of MTM in improving medication non-adherence, the target populations in these studies were those with some specific diseases, such as diabetics (28, 29), osteoporosis (30), HIV/AIDS (31), hypertension (11), and other diseases. In our study, the MTM-eligible beneficiaries were from a general population, had many kinds of diseases, and received different MTM programs. Additionally, there was some evidence that showed no significant improvement in medication adherence in some MTM programs (10, 11).

The literature indicated that the relationship between patients' out-of-pocket medication costs and medication adherence was complex and may be affected by multiple contextual factors. In this population-based study, we also analyzed the independent association between CRN and other covariates such as sociodemographic characteristics, comorbidities, health status, and insurance coverage. We found some variables were also significantly related to CRN. Older people had a lower prevalence of CRN, which may be because they have better care or more care about health issues with age. In terms of gender, women had a higher prevalence of CRN, indicating that there are gender disparities in CRN. Policymakers should issue policies to eliminate the difference between men and women in order to promote healthcare equality. Disparities also reflect geographically, the Medicare beneficiaries living in the South had a higher prevalence rate than the beneficiaries in the Northeast, which may be due to the level of development in different regions and the uneven healthcare supply situation. In terms of socio-economic, compared to low-income beneficiaries (annual income <10,000), those with high income (annual income >40,000) had a significantly lower prevalence of the CRN, while those with middle-income (annual income between 10,001 to 20,000) had a higher prevalence. Meanwhile, beneficiaries without low-income subsidy had a higher prevalence of the CRN, which was consistent with previous study (23, 27). As such, the economic level will affect the CRN, and financial subsidies may reduce the prevalence of the CRN. In any case, a small amount of financial assistance may have unexpected consequences and increase the prevalence. More research is needed in the future to find out what level of financial assistance can most effectively help patients reduce CRN. Because smokers had a higher prevalence of the CRN, it is possible to consider holding educational sessions about quitting smoking to reduce the prevalence of the CRN. In terms of physical health, the worse the health is, the higher the prevalence of CRN. This may be because patients with worse health can not take good care of themselves and non-adherence will partly further result in bad health. However, the causal relationship between the two needs further studies.

This study has a broad implication for health and social policy. It reported the prevalence of CRN among MTM-eligible beneficiaries in a nationally representative sample for the first time. The higher CRN rate in MTM-eligible beneficiaries suggested that these MTM-eligible beneficiaries with a higher estimated annual medical cost, multiple diseases, and medications, may still have problems in affording their medical or medication cost, which may, in turn, lead to bad therapeutic outcomes.

In this study, we also illustrated that MTM eligibility was associated with CRN. That means some strategies should be developed to evaluate the MTM from a comprehensive perspective. To address this issue, CMS is initiating and testing a Medicare Part D Enhanced MTM Model with a five-year performance period that began January 1, 2017. The goals of this model are to learn the best “right-size” for MTM services, to optimize medication use, to improve care coordination, and to strengthen health care system linkages (32). Our study suggests that the identification of alternative strategies to improve CRN should be considered in future Medicare Part D Enhanced MTM Models for better medication management.

There are several limitations in this study. Firstly, we were not able to identify MTM-eligible beneficiaries who used MTM services, which might bias the prevalence of CRN in the MTM users to a certain extent. Secondly, this study used a cross-sectional study design with 1 year of data, which was not able to demonstrate a long-term effect of MTM on CRN, and the causal reference between MTM-eligible beneficiaries and CRN could not be identified. Finally, in this study, because there were no specific identifiers of MTM, we used MTM-eligibility to determine if Medicare beneficiaries participated in MTM programs, which might bring biases to the results. However, according to CMS documentation, patients with MTM-eligibility are identified by Medicare sponsors using claims data and are enrolled in the MTM programs. Therefore, MTM-eligible patients in this study were expected to have already participated in the MTM program and the biases were minimal.

## Conclusion

We found that the prevalence of CRN among MTM-eligible individuals was higher in the MTM group than in the non-MTM-eligible group. MTM was associated with CRN and the odds of CRN increased with participating MTM programs. Understanding the effect of MTM on CRN is essential for comprehensively evaluating and optimizing MTM service. Further studies with the longitudinal design are warranted to clarify the relationship between MTM and CRN. Alternative strategies to improve CRN should be considered in future Medicare Part D Enhanced MTM Models.

## Data Availability Statement

The data analyzed in this study is subject to the following licenses/restrictions: The data will not be made readily available for the privacy of the participants. Requests to access these datasets should be directed to https://www.cms.gov/Research-Statistics-Data-and-Systems/Research/MCBS.

## Ethics Statement

The studies involving human participants were reviewed and approved by Institutional Review Board of the University of South Carolina. Written informed consent for participation was not required for this study in accordance with the national legislation and the institutional requirements.

## Author Contributions

WZ and GL took part in the study design, literature review, and critical draft writing. XX and ML performed data analysis. All authors contributed to the article and approved the submitted version.

## Conflict of Interest

The authors declare that the research was conducted in the absence of any commercial or financial relationships that could be construed as a potential conflict of interest.
